# The role of ferroptosis in virus infections

**DOI:** 10.3389/fmicb.2023.1279655

**Published:** 2023-11-23

**Authors:** Jing Wang, Junda Zhu, Shuning Ren, Zihui Zhang, Kang Niu, Hua Li, Wenxue Wu, Chen Peng

**Affiliations:** National Key Laboratory of Veterinary Public Health and Safety, Key Laboratory of Animal Epidemiology of the Ministry of Agriculture and Rural Affairs, College of Veterinary Medicine, China Agricultural University, Beijing, China

**Keywords:** regulated cell death, ferroptosis, viral infections, virus-host interaction, inhibitors and inducers

## Abstract

Regulated cell death (RCD) is a strategy employed by host cells to defend invasions of pathogens, such as viruses and bacteria. Ferroptosis is a type of RCD characterized by excessive accumulation of iron and lipid peroxidation. While ferroptosis is primarily considered as a mechanism associated with tumorigenesis, emerging evidence begin to suggest that it may play essential role during virus infections. Recent studies illustrated that activation of ferroptosis could either induce or prohibit various types of RCDs to facilitate virus replication or evade host surveillance. More experimental evidence has demonstrated how viruses regulate ferroptosis to influence replication, transmission, and pathogenesis. This review summarizes ferroptosis-related metabolism, including iron metabolism, lipid peroxidation, and antioxidant metabolism. Furthermore, we discuss the interplay between viral infections and host ferroptosis process, with a focus on the mechanism of how viruses exploit ferroptosis for its own replication. Understanding how ferroptosis impacts virus infection can offer valuable insights into the development of effective therapeutic strategies to combat virus infections.

## Introduction

In recent years, there has been a concerning rise in the incidence of emerging and re-emerging viral infections despite the advancements made in preventing and controlling infectious diseases. The occurrence of various epidemics and pandemics caused by viruses has seriously threatened human health, such as severe acute respiratory syndrome coronavirus-2 (SARS-CoV-2) (Choi, 2021), the middle east respiratory syndrome coronavirus (MERS-CoV), the influenza A virus (IAV) (Du et al., 2023), Ebola virus (EBOV) and monkeypox virus (Chen and Whitehead, 2021). Additionally, several animal viruses, such as porcine reproductive and respiratory syndrome virus (PRRSV) (Guo et al., 2021), african swine fever virus (ASFV) (Li et al., 2022), avian influenza virus (AIV) (Spackman, 2020) cause significant economic setbacks to the industry. Although extensive studies have proven that viral infections trigger or evade RCDs, the mechanisms vary among different viruses (Verburg et al., 2022; Wang M. P. et al., 2022). Cell death is a double-edged sword during viral infection (Ashida et al., 2021; Wang M. P. et al., 2022). While virus-associated RCD serves the purpose of expending viral clearance, it may also function to protect the virus from the host defense mechanisms (Jorgensen et al., 2017; Tummers and Green, 2022). Emerging evidence indicates that multiple stages of the virus life cycles may either induce or prohibit RCDs, while the virus proteins responsible for these processes exhibit significant variations (Kerr et al., 2021). Therefore, exploring the mechanisms of cell death in viral infection is vital for developing new therapeutic strategies.

Cell death is an inevitable process that occurs naturally in both physiological and episodic circumstances for all cells, can be categorized into accidental cell death (ACD) and regulated cell death (RCD), which includes genetically induced cell death (Li et al., 2021; Chen et al., 2021a). RCD is a homeostatic mechanism conserved in different species and is necessary to maintain tissue morphology and function (Gao et al., 2022). In animal cells, so far, two primary types of RCDs have been identified, namely apoptotic and nonapoptotic cell death dependent on whether caspases are involved (Koren and Fuchs, 2021). Although apoptosis has been extensively studied, there is a growing body of evidence identifying various non-apoptotic RCDs, such as pyroptosis, ferroptosis, necroptosis, cuproptosis and autophagy-dependent cell death, all of the above have been linked to pathogenic infection (Tang et al., 2019; Koren and Fuchs, 2021; Peng et al., 2022). Among these RCDs, ferroptosis is an iron-dependent cell death that can be trigged by erastin, a small molecule, in cancer cells (Tang et al., 2021; Lei et al., 2022). Mechanistically, it has been demonstrated that ferroptosis distinct from other forms of nonapoptotic and plays vital roles in diseases (Sun et al., 2020). Iron plays essential roles in DNA synthesis and replication, but in excessive conditions, iron can also contribute to the generation of ROS and promoting of lipid peroxidation (Li et al., 2021; Morales and Xue, 2021). Notably, these reactions can be effectively inhibited through the use of glutathione peroxidase 4 (GPX4), iron-chelating agents, and lipophilic antioxidants (Miotto et al., 2020; Jiang et al., 2021). Consequently, disrupting cellular progress may prove to be a valuable and promising therapeutic strategy for viral infections by inhibiting ferroptosis.

Numerous studies have reported the connections between ferroptosis and viral infections. In this review, we summarize the role of iron, antioxidant, and lipid peroxidation in ferroptosis and review the potential mechanism of ferroptosis induction and regulation during viral infection.

### Overview of ferroptosis

The concept of ferroptosis comes from the discovery of selective lethal compounds in RAS-mutant tumor cells (Dixon et al., 2012; Zhao L. et al., 2022). The small molecule, erastin, was the first ferroptosis agent identified in 2003 through a high-throughput small molecule-screening study (Dixon et al., 2012; Zhao et al., 2020). However, the subsequent failed to display sufficient evidence to indicate the targets for erastin-induced cell death (Zhao et al., 2020). The term ferroptosis was coined by Dixon et al. in 2012 to describe iron-dependent cell death (Dixon et al., 2012). It was demonstrated that erastin can induce a new form of cell death that can be alleviated by iron chelators and lipophilic antioxidants, but is immune to apoptotic inhibitors or lysosomal function/autophagy inhibitors. In addition, RSL3, initially identified as RAS-selective lethal (RSL) compound was also capable of activating ferroptosis (Mishima and Conrad, 2022). However, RSL3-induced ferroptosis was independent of voltage dependent anion channel 2 (VDAC2) and voltage dependent anion channel 3 (VDAC3) or cystine/glutamate antiporter (system Xc^−^), indicating the existence of distinct activating routes for ferroptosis (Sun et al., 2020; Yang et al., 2020). GPX4 is reported an essential regulator of ferroptosis through reducing phospholipid hydroperoxide induced by RSL3 in 2014 (Dixon et al., 2012; Yang et al., 2014; Yan et al., 2021). It was not until 2018 when ferroptosis was officially defined as a form of RCD by the Nomenclature Committee on Cell Death (NCCD) (Galluzzi et al., 2018). Ferroptosis is activated by oxidative perturbations in cells, which can be inhibited by iron chelators and lipophilic antioxidants (Galluzzi et al., 2018; Chen et al., 2021d). At present, several compounds were found to be effective modulators of ferroptosis, including inhibitors (e.g., DFO, Fer-1) and inducers (e.g., FIN56, tBOOH) (Conrad et al., 2018).

**Figure 1:**
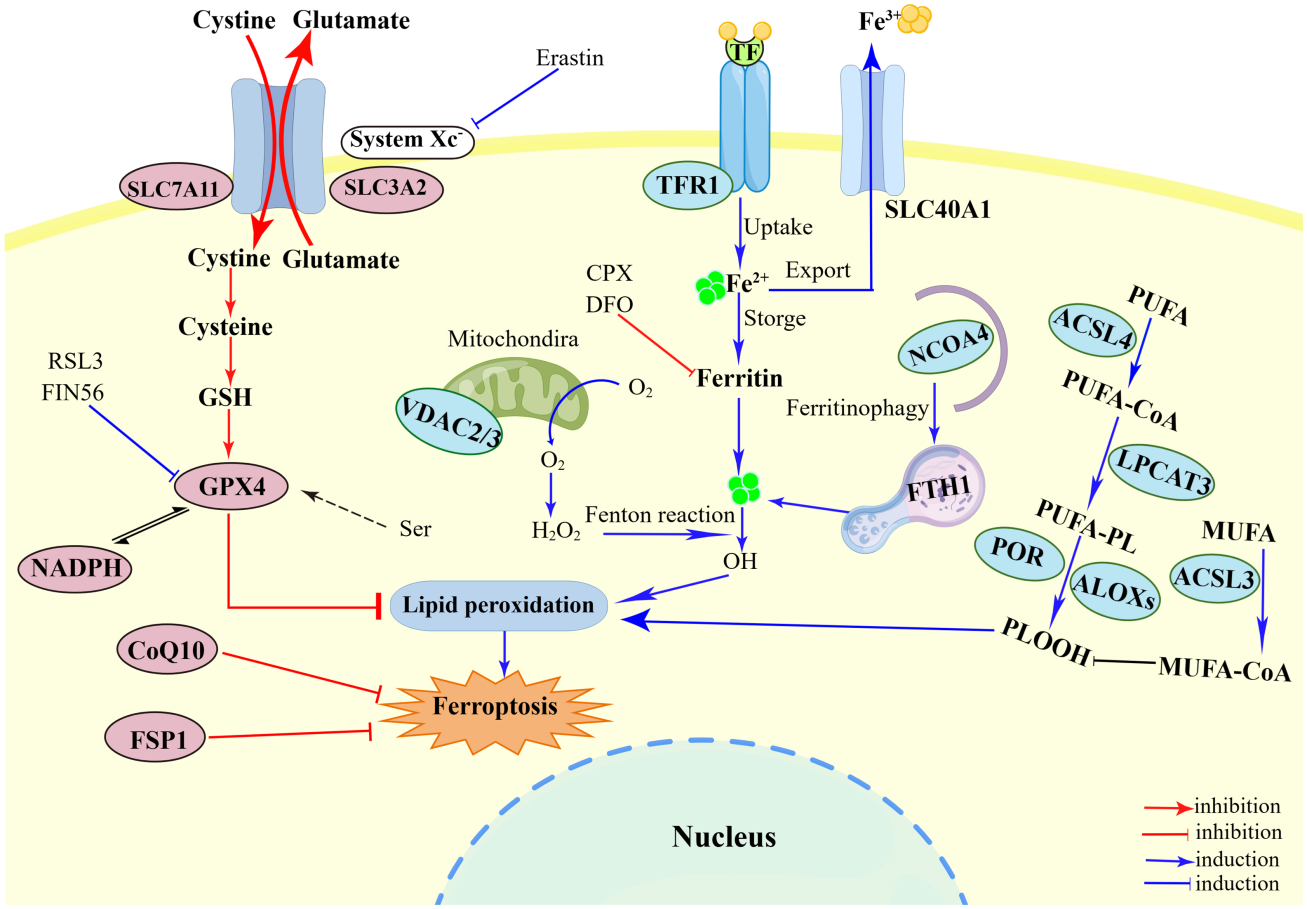
Illustration of the mechanism of ferroptosis. The regulatory of ferroptosis is divided into three parts: iron metabolism, antioxidant metabolism, and lipid metabolism. First, the mechanism of iron metabolism is mainly regulated by NCOA4 pathway and Fenton reaction. Ferritinophagy provides Fe^2+^ to perform lipid peroxidation during the ferroptosis process. The Fenton reaction mediated by Fe^2+^ generates a large amount of HO and promote the peroxidation of PUFA. Second, system Xc^−^-GSH-GPX4, such SLC7A11 inhibition, sulfur transfer pathway, and GPX4 degradation, has act on the antioxidant metabolism. The metabolism of cysteine and reduced GSH constitutes the main line in the ferroptosis pathway. The uptake of cysteine in system Xc^−^ is the upstream event of the ferroptosis reaction under extracellular oxidation conditions, which can be inhibited by erastin. The third part is lipid peroxidation, including ACSL4, LPCAT3, ACSL3, ALOXs, which eventually triggers ferroptosis. PUFAs catalyzed by ACSL4, LPCAT3, and ALOXs converts to PLOOH, and leads to lipid accumulation. Figure was created by Figdraw.

Until now, a growing number of biological markers of ferroptosis have been identified, including morphological, genetic and biochemical hallmarks (Chen et al., 2021a). Ferroptosis has unique morphological features that differ from other RCDs, mainly manifested in mitochondria ultrastructure, manifested as reduction in mitochondrial volume, increase in mitochondrial membrane density, and disappearance of mitochondrial cristae (Li and Li, 2020; Chen et al., 2021c). But the occurrence of ferroptosis can maintain the integrity of plasma membrane and nucleus and without causing concentration of chromatin (Li et al., 2020). Accumulation of cellular iron, as one of the major biochemical hallmarks, plays a crucial role in ferroptosis by elevating Fenton reaction or activating iron-dependent enzymes (Tang et al., 2021; Chen et al., 2021a). Besides, ferroptosis is also characterized by both lipid peroxidation (e.g., isoforms of arachidonate lipoxygenase and PUFA) and activation of antioxidant defense mechanisms (e.g., deleted GSH and activated GPX4) (Chen et al., 2021a). Genetically, ferroptosis is a dynamic process regulated by many genes (e.g., PTGS2, CHAC1 and NFE2L2 genes), which may facilitate lipid peroxidation (Ni et al., 2022). In addition to control by genes mentioned above, the intracellular levels of certain biological marker proteins can be used to assess the process of ferroptosis, such as ACSL4, TFRC, FTH, SLC7A11 and NCOA4 (Xu et al., 2021; Ni et al., 2022). In brief, the regulation of ferroptosis is inseparable from the complex interactions among iron, cysteine and lipid metabolism (Figure 1).

As research has been accumulated, the roles of ferroptosis have been confirmed in various medical conditions such as cancer, osteoporosis, immune response, cardiovascular disease, viral infection, Parkinson’s disease, Alzheimer’s disease, and ischemia–reperfusion injury. These findings underscore the potential significance of targeting ferroptosis as a promising therapeutic approach (Jiang et al., 2021; Liu P. et al., 2022). It is also important to acknowledge that ferroptosis is a cellular process regulated by various regulatory pathways (Dixon and Pratt, 2023).

### Ferroptosis metabolic pathway

#### Iron metabolism

Cellular iron is always in a state of dynamic equilibrium since is regulated by a series of molecular processes (Liang et al., 2022). Iron, as an essential trace element in the body, is involved in the synthesis of proteases, lipid metabolism, as well as overall metabolism (Chifman et al., 2014; Li et al., 2021). Due to its property of reversibly losing or receiving electrons and transferring from one valence state to another, iron catalyzes biochemical reactions, causing cell damage or cell death (Zhou et al., 2020). One of the consequences of is ferroptosis and why this pattern of cell death depends on iron. First, the iron redox may affect sensitivity to ferroptosis. Second, iron can catalyze metabolic enzymes associated with phospholipid peroxidation; and iron is indispensable for the generation of cellular ROS (Fang et al., 2023). Thus, coordinated changes in iron homeostasis regulators affect cellular sensitivity to ferroptosis. The generation of PUFAs-derived lipid peroxidation (e.g., POR and ALOXs) is one of the factors for triggering ferroptosis in the presence of free iron in the cytoplasm (Kuang et al., 2020). These processes are involved in the hydrogen peroxide (H_2_O_2_) and Fe^2+^, named Fenton reaction, which contribute to ferroptosis execution (Bebber et al., 2020). In addition to mediating by Fenton reaction, iron-dependent enzymes are involved in the reaction (Chen et al., 2021c). Several proteins involved in iron metabolism have been proven to modulate ferroptosis, including iron uptake, utilization, storage, and export (Chen et al., 2021b). Extracellular majority circulating Fe^3+^ facilitates the binding of transferrin to its receptor located on plasma membrane (transferrin receptor 1, TFR1) in the form TF-(Fe^3+^)_2_-TFR1, which cause subsequent membrane invagination and formation of specialized endosomes (Guan et al., 2021). After internalization, Fe^3+^ is reduced into Fe^2+^ by endosomal ferrireductase (STEAP3) under acidic conditions, and was transported into the cytoplasm via solute carrier family 11 member 2 (SLC11A2/DMTI) (Chen et al., 2021c; Yan et al., 2021). About 80% of Fe^2+^ in the cytoplasm are stored in the unstable iron pool (LIP) and transferred to mitochondria to synthesize iron–sulfur (Fe-S) clusters (Bayır et al., 2020). Another receptor for TF, TFR2, mainly express in the liver tissue, which has a primary structure similar to TFR1. TFR2 binds TF-bound iron into cells in the same way as TfR1 in a pH-dependent manner (Kawabata, 2019; Tong et al., 2023). However, TFR2 is less-studied compared to TFR1.

In addition, ferritin is the main protein for iron storage located in the cytoplasm, which is composed of a heavy chain (FTH1) and a light chain (FTL) (Park and Chung, 2019). Although iron can be released through the ferritin shells, the main mechanism of iron release is nuclear receptor coactivator 4 (NCOA4)-mediated ferritinophagy (Gryzik et al., 2021). The process involves the direct binding of that NCOA4 to FTH1, resulting in the transportation into autophagosomes for subsequent degradation in lysosomes which ultimately leads to the suppression of ferroptosis (Gao et al., 2016; Gan et al., 2022). The excessive Fe2+ is transported extracellularly by binding to SLC40A1 (also known as FPN1) in mammalian cells (Liang et al., 2019). Three enzymes, ceruloplasmin (CP), Hephaestin (HEPH), and Hephaestin like 1 (HEPHL1), are known to be involved in the iron oxidation in SLC40A1. In summary, these proteins have important regulatory roles in the import, storage, and export of iron, which have significant impact on the susceptibility of ferroptosis.

#### Antioxidant metabolism

The phenomenon of ferroptosis was first found while screening for small molecule compounds used in cancer treatment (Tang et al., 2021). Now, it is known that two small molecule activators, erastin and RSL3, are the antioxidant system inhibitors, which inhibit system Xc^−^ and GPX4, respectively. It plays important roles in understanding antioxidant proteins and inducing ferroptosis (Liu J. et al., 2022). Antioxidant-related pathways regulate the sensitivity of ferroptosis especially the system Xc^−^-GSH-GPX4-dependent antioxidant defense. Besides, the balance of antioxidant metabolism is also maintained by coenzyme CQ10 and dihydroorotic dehydrogenase (DHODH), the GSH-independent manners, which can inhibit ferroptosis (Chen et al., 2021c). GSH is synthesized from glutamate, glycine, and cysteine under the catalysis of the GCL enzyme and GSS enzyme, which are involved in ferroptosis regulation (Doll et al., 2017; Ursini and Maiorino, 2020). Intracellular cysteine, as one of the three amino acids, is notably recognized as a limited factor for the synthesis of GSH, which is mediated by system Xc^−^ (also named glutamate/cysteine antiporter) (Yang and Stockwell, 2016; Chen et al., 2021c). System Xc^−^ consists of SLC7A11/xCT (solute carrier family member 7 member 11) and SLC3A2 (solute carrier family 3 member 2), and is a transporter of glutamic and cystine that promote its transport on the plasma membrane (Aschner et al., 2022). Suppression of system Xc^−^ serves as one of the pivotal factors in ferroptosis activation. A prime example is found in the action of erastin, which is able to hamper the activity of system Xc^−^ and consequently initiates the process of ferroptosis (Battaglia et al., 2020). Studies have shown that inhibition of system Xc^−^ leads to decrease in intracellular GSH levels and facilitates ROS accumulation, eventually resulting in the occurrence of ferroptosis (Pan et al., 2021; Zhao T. et al., 2022). Moreover, the inhibition of SLC7A11 is also an important upstream mechanism for inducing ferroptosis (Koppula et al., 2021a).

Glutathione peroxidase 4 (GPX4), a vital marker for ferroptosis, is an enzyme essential for protecting cells against oxidative damage. GPX4 requires selenium, an essential trace mineral, as a cofactor for its proper function (Conrad and Friedmann Angeli, 2015; Seibt et al., 2019). Selenium is essential for life activities, and its deficiency induce ROS-dependent cell death in serum-free medium (Gan et al., 2022). As an essential amino acid of the GPX4, selenocysteine tRNA (tRNASec) inserts selenocysteine into the UGA codon through a complex process (Yang and Stockwell, 2016; Yan et al., 2021). It has been reported that the mevalonate (MVA) pathway may affect the GPX4 synthesis by modulating the maturation of tRNASec to suppress the occurrence of ferroptosis in cells (Yu et al., 2017; Li et al., 2020). Isopentenyl pyrophosphate (IPP) and coenzyme Q10 (CoQ10), the substrate of enzymatic isopentenylation of tRNASec, suppress ferroptosis through inhibiting lipid peroxidation (Bersuker et al., 2019; Liu J. et al., 2022). Furthermore, FSP1, known as apoptosis inducing factor mitochondria associated 2 gene (AIFM2), is a new limiting factor in ferroptosis by reducing the expression of CoQ10 (Doll et al., 2019). In the process, reduced ubiquinol catalyzes NAD(P)H to capture lipid peroxyl radicals, providing protection against ferroptosis (Chen et al., 2021c).

Importantly, GPX4 is the only number of the GPX protein family that can convert cytotoxic lipid peroxides (L-OOH) to the corresponding alcohols (L-OH) for effective prohibition of ferroptosis (Xu et al., 2021). Indeed, small molecule compounds can block GPX4 pharmacologically, such RAS-synthetic lethal (RSL3), ferroptosis inducer 56 (FIN56), and ferroptosis inducer endoperoxide (FINO2) (Santagostino et al., 2021). GPX4 is an important cofactor in antioxidant metabolism, and its activity and GSH levels can be suppressed by inhibiting system Xc^−^, causing the occurrence of ferroptosis (Seibt et al., 2019).

Thioredoxin (TXN) system is a complex network of proteins and enzymes that participate in the modulation of cellular redox balance and protection of cells against oxidative stress (Lu and Holmgren, 2014; Zheng and Conrad, 2020). A study showed that chemical TXN inhibitor induced ferroptosis in cancer cells, suggesting that the TXN system may act as a negative regulator of ferroptosis in cells (Llabani et al., 2019). It was reported that SLC7A11-GSH-GPX4 axis is an important upstream pathway of ferroptosis, in which the overexpression of SLC7A11 is thioredoxin reductases (TXNRD1)-dependent (Liu X. et al., 2022; Wang Y. et al., 2023). The GCH1/BH4/DHFR axis, and the FSP1/CoQ10 axis are two other important antioxidant axes that are promoted by NADPH (Wei et al., 2020; Liu M. et al., 2022). However, inactivation of GPX4 manifested resistance to ferroptosis in some cancer cell lines, indicating the existence of other defense mechanisms.

#### Lipid peroxidation

Lipid peroxidation is a hallmark of ferroptosis and caused by complex metabolism processes, including Fenton reaction and iron-dependent enzymatic reaction. These reactions directly catalyze the formation of lipid radicals (Li and Li, 2020). Studies have suggested that ferroptosis involves the oxidation of specific polyunsaturated fatty acids (PUFAs) containing phosphatidylethanolamine (Tang et al., 2021). As we all know, lipid is hydrocarbon-containing biomolecules that form the structure and function cell membranes. Lipids are divided into polyunsaturated fatty acids (PUFAs) and monounsaturated fatty acids (MUFAs) depending on its function and source (Chen et al., 2021b). During ferroptosis, PUFAs is the main substrates for lipid peroxidation, especially the arachidonic acid (AA) (Rochette et al., 2022). Free PUFAs are esterified to membrane phospholipids in lipid metabolism, such as phosphatidylethanolamines (PEs) (Tang et al., 2021). PEs must be oxidized into lipid peroxides by ROS within the action of specific enzymes to be effective ferroptosis hallmarks. Acyl-CoA synthetase long-chain family member 4 (ACSL4) lysophosphatidylcholine acyltransferase 3 (LPCAT3) are key enzymes in the PEs biosynthesis and PUFAs activation, finally changing its transmembrane characteristics (Jiang et al., 2021). Therefore, knockout or knockdown of the expression of ACSL4 and LPCAT3 can reduce the accumulation of lipid peroxide in cells, thereby suppressing ferroptosis (Yuan et al., 2016; Doll et al., 2017).

In mammalian cells, although all five isoforms of ACSLs (ACSL1, ACSL3, ACSL4, ACSL5, ACSL6) can selectively activate FAs and convert to acyl-CoAs, its specificity is not strictly depended on substrate preferences (Yuan et al., 2016; Klett et al., 2017). ACSL4 expression is corrected with the sensitivity to erastin-induced ferroptosis, but not other ACSLs forms. ACSL4 is not only a monitoring factor of ferroptosis, but an important regulator of lipid metabolism in such a process (Yang et al., 2022b). Arachidonic acid (AA) and adrenaline (ADA) combine with coenzyme A to catalyze the generation of AA-CoA and ADA-CoA under the action of ACSL4, promoting ferroptosis. Lipid peroxidation can be formed by enzymatic and non-enzymatic radical chain reactions, such as cyclooxygenases (COXs), cytochrome p450s (CYPs), and lipoxygenases (LOXs) (Koppula et al., 2021b). Among these enzymes, LOXs are primarily responsible for the synthesis of lipid hydroperoxides, while COXs and CYPs synthesize lipid endoperoxides and epoxyeicosatrienoic acids (EETs), respectively. LOXs inhibition is effective in preventing ferroptosis by specific inhibitors (e.g., 12/15LOX-ML351, 5LOX-zileuton, tocopherols) (Lv et al., 2022). There are enzymes that promoting ferroptosis by the arachidonate lipoxygenase (ALOX), including ALOX5, ALOX12, ALOX15, ALOX15B and ALOXE3 (Sun et al., 2020). Besides, lipid metabolism can also be regulated in the non-enzymatic pathway, the Fenton reaction (Wang M. P. et al., 2022). Fenton reaction, an oxidation reduction reaction, can transformed hydrogen peroxide (H^2^O^2^) to toxic hydroxyl (HO•) radical through reacting with iron (Fe^2+^ or Fe^3+^), which is an essential process in the oxidation of phospholipids (Kajarabille and Latunde-Dada, 2019). On the contrary, MUFAs activation mediated by ACSL3 displaces PUFAs from phosphatidylethanolamine (Jiang et al., 2021). MUFAs suppress the accumulation of the toxic lipid ROS and the levels of phospholipids to inhibit ferroptosis occurrence (Yang et al., 2022b).

### Ferroptosis and viral infections

#### Iron regulation and viral infections

**Figure 2:**
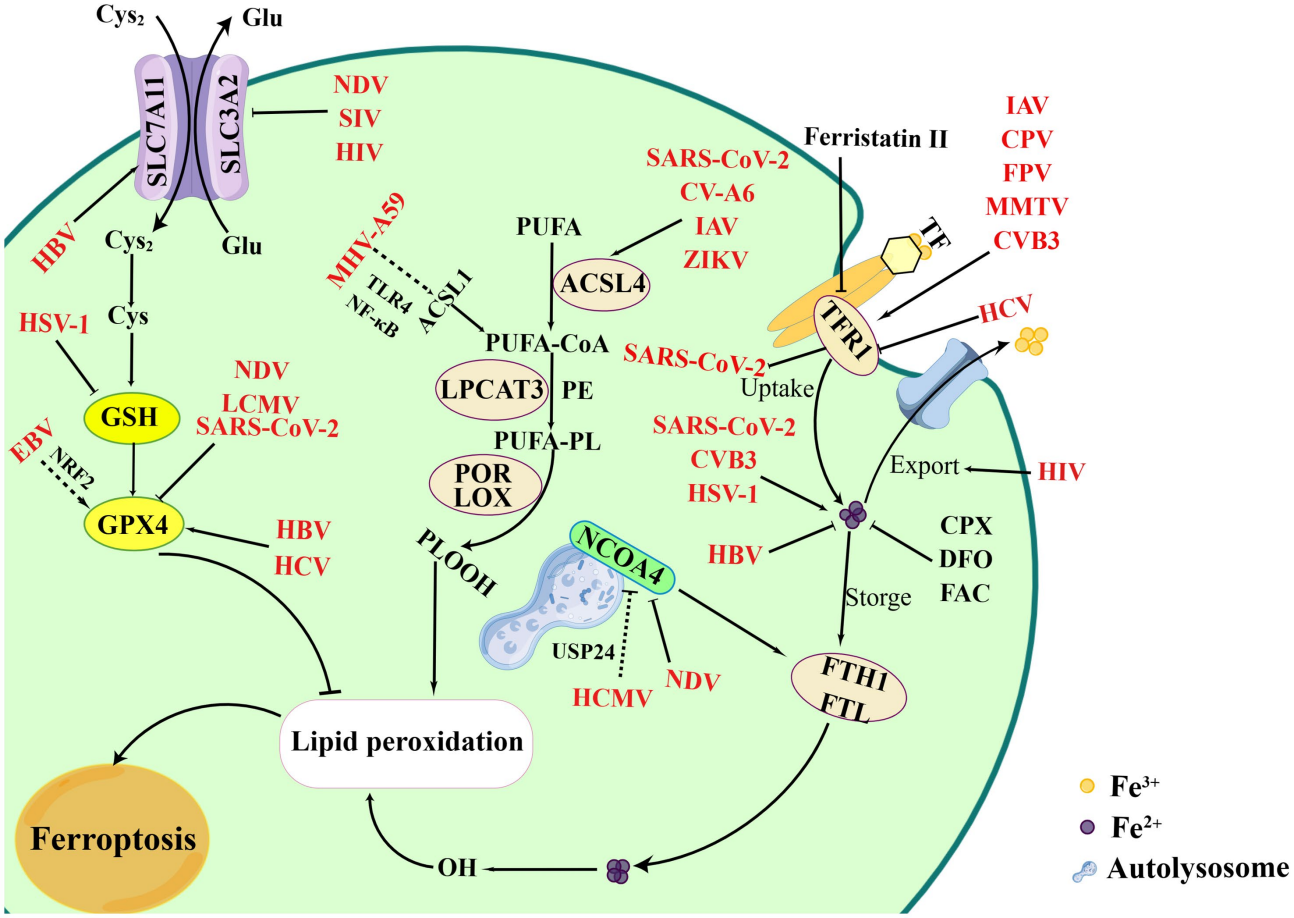
Overview of ferroptosis and viral infections. Viral infections attempt to exploit the process of ferroptosis to benefit its replication. Certain viruses intend to bind TFR1 to entry cells, such as CVB3, IAV, MMTV, CPV, etc., which finally leading to iron accumulation and ferroptosis. However, HCV infections inhibit TFR1 to bypass iron-mediated inhibition. Meanwhile, NDV infection can induce ferritinophagy mediated by NCOA4, while HCMV inhibit the process by combining USP24. Additional processes, including regulation of iron levels, inhibition of iron export, degradation of ferritin protein, can affect viral infections, such as SARS-CoV-2, CVB3, HSV-1, HBV. EBV indirectly damages ferroptosis by promoting NRF2 to inhibit the GPX4. However, SIV, SARS-CoV-2, NDV inducing ferroptosis via suppressing GPX4. In inducing the process of ferroptosis, CV-A6, ZIKV, and IAV activates lipid peroxidation and ferroptosis. MHV-A59 upregulates ACSL1 and induces ferroptosis based on NF-κB and TLR4. Figure was created by Figdraw.

Iron is an essential element to support the cellular basic processes. And it is crucial to support the growth, virulence, and pathogenicity of viruses and microbes, which can obtain iron from the host (Mancinelli et al., 2020). Certain viruses specifically hijack iron-rich cells for replication and alter several mechanisms of iron metabolism (Chhabra et al., 2020). Iron is involved in the regulation of various important biological functions and acts as an essential element for oxygen transfer and serves as both electron donors or acceptors (Vogt et al., 2021). Ferritin is the main site for storing Fe^3+^ which is capable of carrying up to 4,500 iron molecules in its core (Marchetti et al., 2020). Hepcidin, a key protein for iron homeostasis, binds to ferritin and leads to its internalization and degradation (Ginzburg, 2019; Nemeth and Ganz, 2021). A growing number of studies have identified the relationship of viral infections and iron metabolism (Figure 2). For example, the ectopic expression, ferroportin, an iron-regulated protein, could reduce Human immunodeficiency virus (HIV) replication by promoting iron export in PBMCs (Kumari et al., 2016). In addition, SARS-CoV-2 led to iron overload and discharged it into circulation causing ROS-induced oxidative damage and ferroptosis, while the phenomenon could be inhibited by iron chelators. The overexpression of hepcidin and iron overload were promising therapeutic targets for COVID-19, and could be used to measure the effectiveness of treatment and iron homeostasis restoration (Banchini et al., 2020; Habib et al., 2021; Naidu et al., 2023). Cavezzi et al. reported that ferritin was regarded as a typical marker for SARS-CoV-2-induced inflammatory responses, and favored virus replication as a potential pathogenic mediator via ferroptosis. Since the combine role of SA molecules, ACE2, CD147, viroporins and hepcidin induced ferroptosis in the later stages of infection (Cavezzi et al., 2022). These data indicate that chelating iron or altering iron metabolism can prevent SARS-CoV-2 from entering host cells. Intracellular iron overload is known to be a key parameter affecting host infection. However, many viral infections show a reverse consequence between iron increase and hepcidin levels. HCV infections, as an exception, presented the down-regulation of hepcidin and could be up-regulated upon antiviral treatment in the chronic phase. The inhibition of hepcidin led to FPN up-regulation with increase of iron export (Mancinelli et al., 2020).

Iron uptake is indispensable when viral infections cause the intracellular iron overload. TFR1, the receptor of TF, plays an essential role in controlling the iron levels in the cytoplasm that have been involved in viral infections. HCV infection inhibited the expression of TFR1 and DMT1 leading to an iron-deficient phenotype in Huh7.5.1 cells (Fillebeen and Pantopoulos, 2013). Studies also reported that TFR1 played a role at the level of HCV glycoprotein-dependent entry by blocking the cell surface TFR1, and participated in the internalization process acting at the downstream of CD81 in HCVcc and HCVpp infections (Martin and Uprichard, 2013). Moreover, a variety of viruses have been identified to utilize TFR1 for binding and entry the host cells. CVB3 infection increased iron accumulation and TFRC expression, which subsequently aggravated the ferroptosis (Yang et al., 2022a; Yi et al., 2022). Certain viruses have been found to hijack cellular TFR1 and promote virus internalization and by clathrin-mediated endocytosis. For example, TFR1 was shown to be a vital protein in promoting the entry of SARS-CoV-2 facilitating virus endocytosis (Sokolov et al., 2023). As a potential therapeutic target, TFR1 exhibits the effect of inhibiting SARS-CoV-2 infections by chemical inhibitors (such as ferristatin II) (Sokolov et al., 2022; Wang X. et al., 2023). Besides, several viruses have also been characterized to rely on TFR1-dependent entry, including Influenza A virus (IAV), canine parvovirus (CPV), feline panleukopenia virus (FPV), and mouse mammary tumor virus (MMTV) (Kaelber et al., 2012; Martin and Uprichard, 2013; Callaway et al., 2018). Viral particles can enter host cell as cargos by hijacking the TFR1-mediated endocytic pathway, enabling pH-dependent membrane fusion (Mazel-Sanchez et al., 2023). Because the TFR1 receptor is redirected back to the cell surface, multiple rounds of infection and superinfection could occur through the use of TFR1 (Wessling-Resnick, 2018).

NOCA4, a selective cargo receptor, promotes ferritin degradation during ferritinophagy and contributes to the occurrence of ferroptosis (Tang et al., 2018). Previous studies have shown that Newcastle disease virus (NDV) promoted viral replication by triggering autophagy. However, the latest research showed that NDV infections reduced the expression of NCOA4 and FTH1, which led to the release of ferrous irons and induce ferroptosis as a result of accumulation of lipid ROS (Kan et al., 2021; Gao et al., 2023). This result provided promising insights for the study of oncolytic viruses to improve oncolytic effects for those therapy-resistant cancers. Sun et al. reported that Human cytomegalovirus (HCMV) protein pUL38 blocked the function of USP24 to prevent iron-dependent cell death. USP24 regulated and stabilized the protein levels of NCOA4 thereby promoting ferritinophagy during HCMV infection, while the binding of USP24 to pUL38 was able to reduce ferritinophagy to promote iron-dependent ER stress-induced cell death (Santana-Codina and Mancias, 2018; Sun et al., 2018). Regulating NCOA4-mediated ferritinophagy levels may also be an effective strategy to suppress certain viral infections.

Overall, iron is abundantly present in mammals, either existing freely or bound to hepcidin. It exhibits the ability to modulate the replication of various viral infections across different organisms. The main pathways by which ferroptosis affects viral replication are the iron transport by iron-related proteins and autophagy degradation pathways. The regulatory mechanisms involved extend beyond the impact of iron-related genes, and involved the action of small molecules. Notably, certain small molecules such as DFO, FAC, ciclopirox olamine have been well-documented to possess antiviral activity.

#### Antioxidants and viral infections

Selenium plays an essential role in redox signaling, antioxidant defense and redox homeostasis. The important role of selenoprotein members has been demonstrated during viral cycle including GPX and TXNRD (Guillin et al., 2019). Therefore, viral infections also shown to be regulated by antioxidants. In antioxidant metabolism, viruses may benefit greatly in GPX4-dependent and SLC7A11-dependent manner, causing increase of viral proliferation or pathogenesis. Yuan et al. suggested that Epstein–Barr virus (EBV) reduced the sensitivity of ferroptosis by activating p62-Keap1-NRF2 pathway and upregulating GPX4 and SLC7A11 expression in NPC cells (Yuan et al., 2022). H1N1 swine influenza virus (SIV) infection disrupted iron uptake and storage to enhance viral replication through inhibiting the system Xc^−^/GPX4 axis (Cheng et al., 2022). These results demonstrated that GPX4 modulated by ferroptosis was a promising novel target for the treatment of viral diseases. GSH deficiency led to GPX4 inactivation, thereby inducing ferroptosis due to the accumulation of lipid-free radicals (Zhang et al., 2021; Wang M. P. et al., 2022). This phenomenon was initially manifested in cancer and injury, but also in viral infections, such as HIV (Song et al., 2016; Yang W. et al., 2022). Studies found that HSV-1 infections induced ferroptosis demonstrated by iron overload, ROS accumulation, GSH deletion, and lipid peroxidation (Richard et al., 2001; Xu et al., 2023). Moreover, the antiviral effect of GPX4 was found in HBV, HCV and SARS-CoV-2 infections (Brault et al., 2016; Hu et al., 2022). It is noteworthy that GPX4 also played an important role in innate immunity, manifesting by immune responses to infection caused by acute lymphocytic choriomeningitis virus (LCMV) (Matsushita et al., 2015; Wang Y. et al., 2022).

As mentioned above, the relationship between viral infections and the system Xc^−^/GSH/GPX4 axis is complex. Depending on the different virus, altering the axis plays distinct roles in its replication. These studies suggest that GPX is critical in viral infection and may be a target for antiviral research.

#### Lipid metabolism and viral infections

Lipids are a major and diverse class of biomolecules that play important roles in the physiology and pathophysiology. The role of lipids in viral infections has been reported since altering lipids of host cells to enhance their viral life cycle (Monson et al., 2021). And its roles display in the fusion of viral membranes to host cells, viral replication, and viral endocytosis and exocytosis (Farías et al., 2022). Viral infection can induce ferroptosis by lipid ROS synthesis in various mechanisms, such through the function of ACSL4. ACSL4 promotes ferroptosis by producing 5-HETE-mediated lipotoxicity, which is found to be overexpressed in various kinds of cancer cells. An example is that enteroviruses (CV-A6) was able to induce ferroptosis through an increase in lipid peroxidation. Kung et al. suggested ACSL4 was involved in viral replication cellular organelle formation, including the endoplasmic reticulum (ER) and Golgi membranes (Kung et al., 2022). In viral infection, ACSL4 exhibited the similar promoting effect for SARS-CoV-2, IAV, and Zika virus (ZIKV), demonstrating a unifying mechanism of lipid peroxidation in virus infections. The most important is that ferroptosis inhibitors, rosiglitazone (ROSI) and pioglitazone (PIO), reduce coronaviruses load (Kung et al., 2022). The study revealed a novel mechanism by which altering or targeting lipid peroxidation may block viral replication.

Additionally, ACSL1, a novel ferroptosis inducer, was shown proved to promote PUFA-PL production and ROS synthesis (Xia et al., 2021). Murine hepatitis virus strain A59 (MHV-A59) induced ferroptosis in primary macrophages by upregulating the expression of ACSL1. Ferroptosis inhibitor (liproxstatin-1) and ACSL1 inhibitor (Triacsin C) could protect cells from infection. They also explained that the results were dependent on NF-κB and the toll-like receptor 4 (TLR4) pathways (Xia et al., 2021). ACSL1 may be possibly a novel therapeutic target to limit inflammation in COVID-19 or other viral infections.

Notably, it was reported that PUFA-PE was related with lung injuries and bacterial infection (e.g., *P. aeruginosa*) (Dar et al., 2022), however, its role in viral infections remained unclear. Until now, the relationship between viral infections and lipid peroxidation in ferroptosis was not fully understood and necessitated further investigation. ACSL1 and ACSL4 inhibitions prevent ferroptosis during viral infections, thereby protecting the host.

### Effect of viral infections through ferroptosis

The process of ferroptosis is regulated by a series of cellular metabolic pathways, including antioxidant regulation system, iron homeostasis, lipid overload, and other related signaling pathways. All the pathways have been shown to play a vital role in viral infections. Triggering ferroptosis would be advantage to diseases by clearing cells with potential problems. Thus, ferroptosis has been used as a therapeutic measure in many areas.

#### Ferroptosis inducers and viral infections

Until now, ferroptosis inducers (FINs) can be categorized into four informs based on the targets and mechanisms. Class I FINs (e.g., erastin and sorafenib) are inducers that block the system Xc^−^. They reduce the level of GSH and indirectly inhibit GPX4 expression (Xie et al., 2022). Class II FINs (e.g., RSL3) can directly inhibit GPX4 and reduce its activity. Class III FINs (e.g., FIN56) are the inhibitors of GPX4 protein and CoQ10 resulting the accumulation of lipid peroxidation. Class IV FINs (e.g., FINO2) initiates ferroptosis through GPX4 inactivation and iron oxidation. FINs may be useful as the treatment of certain cancer including gastric cancer, lung cancer (Koppula et al., 2021a). For example, erastin would facilitate the role of an oncolytic vaccinia virus that improving the efficacy of cancer immunotherapy (Liu W. et al., 2022). However, few studies have been identified the role of the FINs in viral infections. Zhang et al. demonstrated that erastin could inhibit porcine epidemic diarrhea virus (PEDV) replication in Vero cells (Zhang et al., 2023). RSL3 inhibited HSV-1-induced innate antiviral immune responses through inactivating GPX4, causing the promotion of its replication (Jia et al., 2020). No studies are reported the role of FINO2 or FIN56 and viruses.

#### Ferroptosis inhibitors and viral infections

Ferroptosis inhibitors can be used in cancers and other diseases as well as FINs. Inhibition of ferroptosis is manifested in the blockade of lipid peroxidation. So far, most inhibitors are iron chelators and lipophilic radical-trapping antioxidants (RTAs). For example, iron chelators are divided into membrane permeability (e.g., ciclopirox and 2, 2-bipyridyl) and membrane impermeability (e.g., DFO), which preventing the cells from ferroptosis. RTAs can prevent the chain-propagating peroxyl radical from oxidizing during lipid peroxidation, such as Fer-1 and liproxstatin-1 (Shaghaghi et al., 2022). As FDA-approved compound used to suppress fungal infections, ciclopirox have been shown to inhibit HBV replication through blocking capsid assembly in cells and in a humanized liver mouse model. And the compound works synergistically with TDF and ETV to add effects (Kang et al., 2019). Furthermore, ciclopirox also can inhibit HSV-1, HPV, HIV-1, and HCV (Bernier and Morrison, 2018; Braun et al., 2020; Zangi et al., 2022). The potential to repurpose ciclopirox for new antiviral indications is an exciting prospect. Additionally, DFO is able to inhibit SARS-CoV-2 infection and block SARS-CoV-2-induced ferroptosis (Han et al., 2022). In the above description, SIV could trigger ferroptosis and promote its replication, while the process would be suppressed by Fer-1 (Cheng et al., 2022). Some synthetic radical scavengers (e.g., α-tocopherol) inhibit ferroptosis. Vitamin E (α-tocopherol) both specifically destroy peroxide chain proliferation and inhibit lipoxygenase, which can protect drug-induced liver injury (Maiorino et al., 2018).

In summary, both FINs and ferroptosis inhibitors may be potential for diseases treatment. However, the mechanisms between ferroptosis compounds and viral infections still remain to be explored. Some inhibitors can effectively inhibit ferroptosis, while its potential toxicity has limited their use. To solve this problem, researchers further research need to be conducted in the future.

## Conclusion and perspectives

RCDs have been identified as host defense strategies to restrict viruses. However, the mechanisms by which RCDs affects viral pathogenesis is distinct and limited. At the organismal level, a variety of cellular pathways activation and inhibition may affect virus replication, for example, inflammatory response caused by pyroptosis and necroptosis causes; lipid peroxidation induced by ferroptosis (Verburg et al., 2022). Thus, activation of RCDs must be carefully balanced for the optimal host defense. The latest studies showed ferroptosis, a newly discovery RCD, may play complicated but important role during viral infections. Virus-induced ferroptosis were manifested by several common phenomenon, including the increase of intracellular iron and ROS, as well as GPX4 activity reduction, all of which may disrupt normal cellular metabolism (Wang M. P. et al., 2022). However, emerging evidence has demonstrated that the induction mechanism and process can vary depending on the specific virus, and it has observed that not all viruses were able to trigger ferroptosis.

Viruses frequently hijack or destroy machineries in host cells, which may cause cell death (Table 1). Generally, most viruses exploit ferroptosis to benefit their replication and release, which could facilitate viral transmission and lead to host organ damage, examples of these viruses include but were not limited to HIV and SARS-CoV-2. Moreover, oncolytic viruses may utilize the pathway for ferroptosis to eliminate tumor cells. Recent findings illustrated that ferroptosis is not only a promising target for the treatment of cancers, but also for viral infections including those caused by HCV, IAV, CV-A6, CVB3, and HCMV (Gao et al., 2023). Therefore, further research in the mechanism by which ferroptosis regulated viral infections will provide new insights into the formulation of novel therapeutic measures.

**Table 1:** List of the mechanisms between the viruses and ferroptosis.

Viruses	Interactions	Targeted cells	Outcomes	References
Human immunodeficiency virus (HIV)	Increases iron export mediated by ferroportin to inhibit HIV replication BRD4/miR-29 system opposes ferroptosis by boosting SLC7A11 and ferritinophagy	PBMCs, THP-1	Reduce virus replication	Kumari et al. (2016) and Sfera et al. (2022)
			Promote HIV-induced neurodegeneration	
Herpes simplex virus 1 (HSV-1)	Nrf2 disturbs cellular redox homeostasis to promote ferroptosis	U373	Alleviate HSV-1 encephalitis	Xu et al. (2023)
Severe acute respiratory syndrome coronavirus-2 (SARS-CoV-2)	Decrease GPX4 expression to induce ferroptosis ORF3 induce ferroptosis via Keap1-NRF2 axis Promote expression and internalization of TfR1	Vero, NCI-H1299	Not clear	Jankauskas et al. (2023), Liu et al. (2023), and Wang X. et al. (2023)
Murine hepatitis virus strain A59 (MHV-A59) Hepatitis C Virus (HCV)	Upregulate ACSL1 dependent on NF-κB	RAW264.7, 17CL-1, MEF Huh7.5.1	Prevent viruse infection	Xia et al. (2021)
	Reduce TFR1 and DMT1 expression Induce GPX4 expression by NS5A		Inhibit viruse infection	Fillebeen and Pantopoulos (2013) and Brault et al. (2016)
Coxsackievirus B3 (CVB3)	Induce ferroptosis by regulating the Sp1/TFRC/Fe axis	Hela	Not clear	Yi et al. (2022)
Swine influenza virus (SIV)	Inhibit system Xc^−^/GPX4 axis and increase TFR1 expression	A549	Enhance virus replication	Cheng et al. (2022)
Influenza A virus (IAV)	Increase TFR1 expression to promote IAV infection	A549	Enhance IAV infection	Mazel-Sanchez et al. (2023)
Feline panleukopenia virus (FPV)	Glycosylation site Mutation of TFR1	CCL64	Protect against FPV infection	Callaway et al. (2018)
Newcastle disease virus (NDV)	Induce ferroptosis via p53-SLC7A11-GPX4 pathway	U251	Reduce cancer cell activity	Kan et al. (2021)
Human cytomegalovirus (HCMV)	USP24 combined with pUL38 regulate NCOA4 to reduce ferritinophagy	HEK293T	Not clear	Sun et al. (2018)
Enteroviruses (CV-A6)	Induce ferroptosis by ACSL4	RD, HEK293T, A549	Facilitate virus replication	Kung et al. (2022)
Epstein–Barr virus (EBV)	The upregulation of GPX4 and SLC7A11 induce ferroptosis via p62-Keap1-NRF2; GPX4 interact with TAK1-TAB1/TAB3 Complex to regulate TAK1acticity	HEK293T	Not clear	Yuan et al. (2022)
Lymphocytic choriomeningitis virus (LCMV)	mTORC2 interrupt virus-specific memory CD4+ T cells; mTORC2 inactivation leads to reduce GPX4 expression by the mTORC2-AKT-GSK3β axis	BMCs	Not clear	Wang Y. et al. (2022)

Furthermore, inducers and inhibitors contribute to elucidate metabolic processes and mechanisms. Several studies demonstrated that ciclopirox olamine inhibited viral replication by reducing the intracellular iron. Recently, it is reported that artemisinin banded covalently to intracellular Fe^2+^ for the early evaluation of anticancer drug-induced ACI and AKI (Zeng et al., 2023). These findings contributed to the better understanding of the regulatory processes of ferroptosis and further investigation may contribute to the development of novel antiviral drugs.

## Author contributions

JW: Software, Writing – original draft, Investigation. JZ: Investigation, Writing – review & editing. SR: Investigation, Writing – review & editing. ZZ: Investigation, Writing – review & editing. KN: Investigation, Writing – review & editing. HL: Investigation, Writing – review & editing. WW: Supervision, Writing – review & editing. CP: Supervision, Writing – review & editing.
